# The immune system in sporadic inclusion body myositis patients is not compromised by blood-flow restricted exercise training

**DOI:** 10.1186/s13075-019-2036-2

**Published:** 2019-12-18

**Authors:** Kasper Yde Jensen, Mikkel Jacobsen, Henrik Daa Schrøder, Per Aagaard, Jakob Lindberg Nielsen, Anders Nørkær Jørgensen, Eleanor Boyle, Rune Dueholm Bech, Sofie Rosmark, Louise Pyndt Diederichsen, Ulrik Frandsen

**Affiliations:** 1 Department of Sports Science and Clinical Biomechanics, SDU Muscle Research Cluster (SMRC), University of Southern Denmark, Odense, Denmark; 2 Department of Rheumatology, Odense University Hospital, Odense, Denmark; 3 Department of Pathology, Odense University Hospital, Odense, Denmark; 4 Department of Clinical Research, University of Southern Denmark, Odense, Denmark; 5 Department of Orthopaedics and Traumatology, Copenhagen University Hospital, Rigshospitalet, Copenhagen, Denmark; 6 Center for Rheumatology and Spine Diseases, Copenhagen University Hospital, Rigshospitalet, Copenhagen, Denmark; 7 Department of Sport Science and Clinical Biomechanics, Research Unit of Clinical Biomechanics, University of Southern Denmark, Odense, Denmark

**Keywords:** Sporadic inclusion body myositis, Inflammation, T lymphocytes, T cells, Macrophages, Disease progression, Blood flow restriction exercise

## Abstract

**Background:**

Sporadic inclusion body myositis (sIBM) is clinically characterised by progressive proximal and distal muscle weakness and impaired physical function while skeletal muscle tissue displays abnormal cellular infiltration of T cells, macrophages, and dendritic cells. Only limited knowledge exists about the effects of low-load blood flow restriction exercise in sIBM patients, and its effect on the immunological responses at the myocellular level remains unknown. The present study is the first to investigate the longitudinal effects of low-load blood flow restriction exercise on innate and adaptive immune markers in skeletal muscle from sIBM patients.

**Methods:**

Twenty-two biopsy-validated sIBM patients were randomised into either 12 weeks of low-load blood flow restriction exercise (BFRE) or no exercise (CON). Five patients from the control group completed 12 weeks of BFRE immediately following participation in the 12-week control period leading to an intervention group of 16 patients. Muscle biopsies were obtained from either the m. tibialis anterior or the m. vastus lateralis for evaluation of CD3-, CD8-, CD68-, CD206-, CD244- and FOXP3-positive cells by three-colour immunofluorescence microscopy and Visiopharm-based image analysis quantification. A linear mixed model was used for the statistical analysis.

**Results:**

Myocellular infiltration of CD3^−^/CD8^+^ expressing natural killer cells increased following BFRE (*P* < 0.05) with no changes in CON. No changes were observed for CD3^+^/CD8^−^ or CD3^+^/CD8^+^ T cells in BFRE or CON. CD3^+^/CD244^+^ T cells decreased in CON, while no changes were observed in BFRE. Pronounced infiltration of M1 pro-inflammatory (CD68^+^/CD206^−^) and M2 anti-inflammatory (CD68^+^/CD206^+^) macrophages were observed at baseline; however, no longitudinal changes in macrophage content were observed for both groups.

**Conclusions:**

Low-load blood flow restriction exercise elicited an upregulation in CD3^−^/CD8^+^ expressing natural killer cell content, which suggests that 12 weeks of BFRE training evokes an amplified immune response in sIBM muscle. However, the observation of no changes in macrophage or T cell infiltration in the BFRE-trained patients indicates that patients with sIBM may engage in this type of exercise with no risk of intensified inflammatory activity.

## Introduction/background

Sporadic inclusion body myositis (sIBM) is an acquired slowly progressing inflammatory myopathy with a late age onset (61–68 years of age) [1]. sIBM is characterised clinically by muscle weakness and atrophy, prominently observed in the quadriceps muscles and in the wrists and fingers [2, 3]. In histological terms, sIBM involves inflammatory infiltrates, muscle fibre damage and cytoplasmatic fibrillary inclusions. Over time, the affected muscles accumulate degenerative changes that are dominated by fatty infiltration and muscle atrophy [4, 5]. In result, patients experience a marked progressive decline in muscle strength and physical function. It has been reported that the annual decline in muscle strength averages between 5 and 16% per year [6, 7]. This corresponds to a 5 to 10-fold faster decline than in healthy adults of comparable age [8, 9]. Unlike other subgroups of idiopathic inflammatory myopathies (IIMs), most sIBM patients are non-responsive to immunosuppressive medication [10].

Exercise-based interventions have been documented to induce skeletal muscle hypertrophy, increase contractile strength and to negate/attenuate inflammation in IIMs [11–14]. Therefore, clinical recommendations concerning physical activity in IIMs have gradually changed from avoiding exercise and limiting physical activity, to conversely encourage IIM patients to engage in exercise participation.

Exercises combining low-load muscle contractions and partial venous blood-flow restriction (blood-flow restricted exercise, BFRE) may be particularly relevant to individuals with profound muscle weakness, such as sIBM patients because of its potential ability to stimulate skeletal muscle growth and its potential anti-inflammatory properties [15–17]. Previous case studies and case series have successfully implemented BFRE in sIBM, polymyositis (PM) and dermatomyositis (DM) patients [18–20].

Using a randomised controlled study design, we were unable to verify a direct performance enhancing impact of BFRE intervention in a group of frail sIBM patients [21]. However, when compared to care-as-usual sIBM patients (CON), BFRE training appeared effective of reducing the rate of decline in lower limb muscle strength for this patient group [21].

To further understand the underlying pathophysiological mechanisms of the effects of BFRE in sIBM patients, this study investigated the adaptive and innate immune response of the affected muscles elicited during the 12 weeks of low-load BFRE training.

T cells are an important part of the adaptive immune system, particularly in relation to sIBM with various subgroups being of high relevance. Specifically, CD28^null^ T cells have been shown to be pro-inflammatory and cytotoxic in nature and to increase in density in patients with sIBM compared to age-matched controls [22–26].

Tissue tolerance towards T cell immune infiltration is modulated by regulatory T cells (T_reg_) residing locally within the skeletal muscle tissue. T_reg_ cells maintain immunological protection against self-antigens via inhibition of T cell activation and weakening of T cell effector function [27, 28]. Specifically, in IIM patients, T_reg_ (FOXP3^+^) cells serve to counterbalance muscle destruction caused by cytotoxic (CD8^+^) T cells [29]. Furthermore, T_reg_ cells are known to coordinate immune cell interactions involved in skeletal muscle growth and regeneration [30, 31], by regulating the transition from M1-biased to M2-biased macrophage phenotypes [32]. The reduced expression of T_reg_ cells observed in sIBM patients [29, 33] combined with high numbers of CD28^null^ T cells may facilitate autoimmune-like responses in sIBM muscle tissue [24].

Previous reports have shown that macrophages play a significant role in the recovery from muscle damage and repair while further facilitating myofibre growth [32, 34, 35]. Further, in healthy young adults, we recently observed that BFRE training can induce an upregulation in both pro- (M1) and anti-inflammatory (M2) macrophage content [16]. Natural killer (NK) cells represent a similarly important element of the innate immune system. Specifically, NK cells may be considered the innate immune system’s counterpart to cytotoxic CD8^+^ T cells in the adaptive immune system and are known to increase in response to both acute and chronic exercise [36, 37].

The aim of the present study was to investigate the effects of longitudinal BFRE training on the expression of key immune cell populations involved in sIBM disease progression, skeletal muscle growth and regeneration. It was hypothesised that BFRE training would decrease the expression of cytotoxic T cell infiltrates, increase T_reg_ infiltrates and enhance M1- to M2-biased macrophage transition in trained muscles. In turn, this would contribute to previously explained observations of retained muscle function following BFRE training in sIBM [21].

## Material and methods

### Study design and experimental procedures

**Fig. 1:**
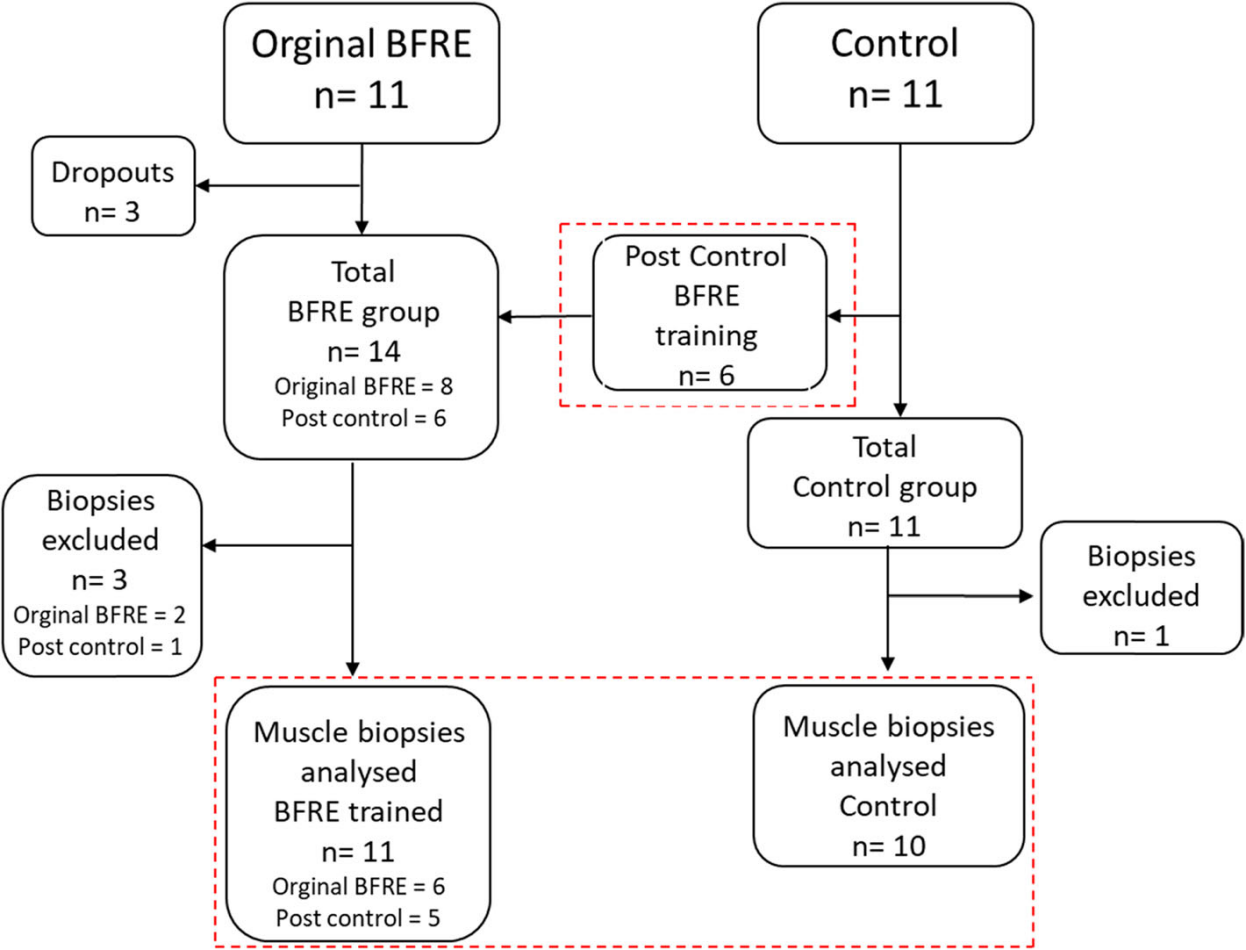
Flowchart of included patients (original BFRE and control). Post control, patients from the control who underwent the BFRE training intervention after the 12-week control period

The study was a randomised controlled trial (RCT). Recruited patients were randomly allocated to the intervention group (BFRE) or the control group (CON), by using a 1:1 random allocation ratio design. Recruitment procedures, testing and training of patients have been described in detail elsewhere [38]. In brief, a total of 22 biopsy-validated [3] sIBM patients (Table 1) were recruited (for detailed overview of study design, see Fig. 1).

**Table 1:** Baseline characteristics of included patients. *BMI* body mass index, *MMT8* manual muscle testing 8

	Age	Gender	BMI	Months from diagnosis	Creatine kinase	Health assessment questionnaire	MMT8	Immunosuppressive medicine
BFRE (*n* = 11)								
Mean	67.5	W/M: 3/8	24.71	57.8	549	0.85	69.6	4/11
SD	6.5		3.19	87.7	397	0.83	6.2	
Control (*n* = 10)								
Mean	69.2	W/M: 2/8	24.69	46.6	308	1.125	67.9	2/10
SD	4.6		4.68	27.2	247	0.88	6.0

### BFRE protocol

The BFRE training protocol has been described in detail elsewhere [38]. In brief, the exercise protocol consisted of low-load (~ 25RM) blood-flow occluded resistance training performed two times per week for 12 weeks, involving five different lower extremity exercises (three to four sets of each exercise).

Vascular occlusion of the lower limb was controlled by an inflatable pneumatic cuff (100-mm width) placed proximally at the thigh or calf. The cuff was connected to a computerised tourniquet system (Zimmer ATS 750, Zimmer, Inc., Warsaw, USA) that automatically regulated the cuff pressure (110 mmHg) [39].

### Muscle biopsy sampling

Muscle biopsies were obtained from m. tibialis anterior or m. vastus lateralis during local anaesthesia, and sterile conditions provided by an experienced medical doctor using a Bergström needle. The muscle samples immediately were embedded in Tissue Tek (4583, Sakura Finetek, Alphen aan den Rijn, The Netherlands) and frozen in nitrogen-cooled 2-methylbutane.

### Immunofluorescence

Transverse serial sections (8 μm) of the embedded muscle biopsies were cut in a cryostat at − 22 °C (HM 560 Cryo-Star Cryostat, Microm, Walldorf, Germany) and placed on SuperFrost plus glass slides (Thermo Fisher, Rockford, USA). The biopsy cryosections were fixed in 4% formaldehyde fixation solution containing 100 μL of Triton X-100 (10%) (Sigma-Aldrich, St. Louis, MO, USA), 200 μL of formaldehyde (37%) and 1.7 mL phosphate-buffered saline (PBS, × 10) (70013, Invitrogen, Paisley, UK) for 10 min. Sections were rinsed for three cycles of 3 min followed by Protein Block (X0909, DakoCytomation, Glostrup, Denmark) for 10 min.

Primary antibodies were incubated overnight at 4 °C. Standard secondary antibodies were incubated for 60 min at room temperature, whereas the AB 488 Vector Mouse enhancer kit was used according to manufacturer’s instructions (Vector, Burlingame, USA) (see Table 2 for a detailed listing of the antibodies used). Antibody incubation was followed by a wash step procedure by using PBS, and every antibody cycle (primary and secondary) was followed by wash step and protein blocking.

**Table 2:** Overview of antibodies and incubation times

Antibody	Origin	Target	Company	Catalogue number	Dilution	Incubation
CD3	Rabbit	T cells	Dako/Agilent	A0452	1:2000	Overnight
CD8	Mouse	T cells and NK	Dako/Agilent	M7103	1:100	Overnight
CD68	Mouse	CD68^+^ macrophages	DakoCytomation	M0814	1:400	Overnight
CD206 (MMR)	Goat	CD 206^+^ macrophages	DakoCytomation	AF2534	1:400	Overnight
CD244	Goat	CD28null T cells	R&D Systems	AF1039	1:100	Overnight
DAPI		DNA	Thermo Fisher	62248	1:10000	10 s
FoxP3	Mouse	Treg cells	Thermo Fisher	11-4777-82	1:500	Overnight
Laminin	Rabbit	Basal membrane	DakoCytomation	Z0097	1:2000	Overnight
MHC—fast	Mouse	Type 2 fibres	Sigma-Aldrich	M4276	1:1000	30 min

After completion of the last antibody cycle, DAPI (62248, Thermo Fisher, Rockford, USA) was placed on the sections and washed off after 5 s. Sections were mounted with Vector AQ mounting medium (H-5501, Vector, Burlingame, USA).

**Fig. 2:**
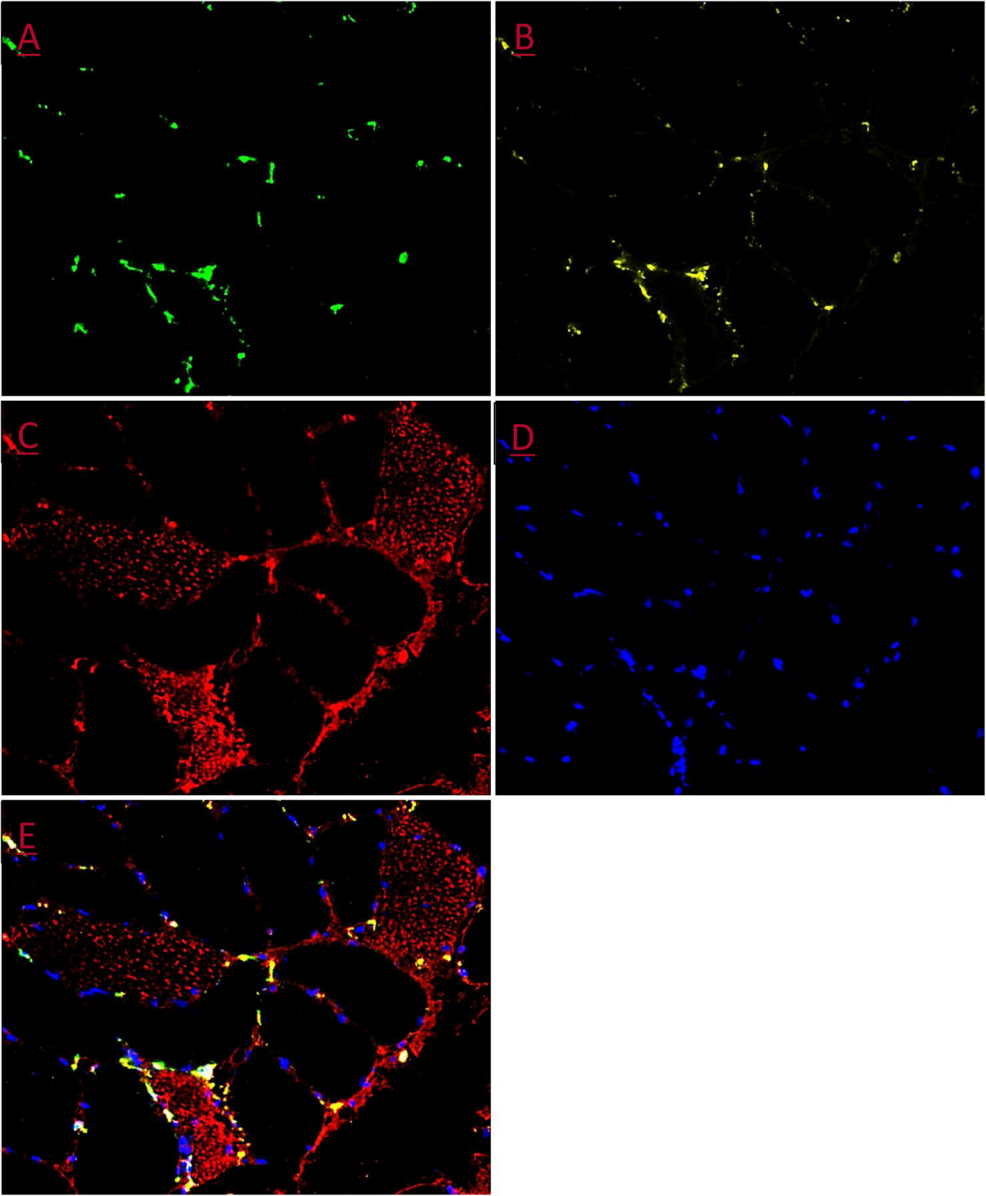
Representative immunohistochemical staining. **a** CD-68. **b** CD-206. **c** MHC-Fast. **d** Nuclei/DAPI. **e** Merged

Stainings were visualised using a light microscope (Carl Zeiss Axio Imager M1, Germany) with a high-resolution AxioCam (Carl Zeiss, Germany) (see Fig. 2 for representative immunohistochemical stainings).

### Data processing

All images were analysed using Visiopharm image analysis software (Visiopharm, Hoersholm, Denmark). Images were analysed using the following three-step protocol:

1. Identify and mark the area containing longitudinal and cross-sectional muscle fibres.
2. Identify immunological markers for T cells, NK’s, M1, and M2 macrophages, and write the script that enables Visiopharm to correctly identify and quantify (i.e. number of immune-positive cells) the respective antibody markers. This methodology was verified by manual counting performed by an experienced researcher.
3. Normalise the number of positive cells to the analysed area (counts/mm^2^).
4. Across the BFRE and CON biopsies, an average area of 3.81 ± 2.01 and 3.07 ± 1.76 mm^2^ was analysed, respectively.

All data analysis was performed by an investigator blinded with respect to patient ID, group allocation and time-point.

### Statistical analysis

A linear mixed model was used to evaluate if there was a within- and between-group change in the selected immune response markers. The model was adjusted for age, months since diagnosis and whether on immunosuppressive  medication or not.

Statistical significance level was set at *p* = 0.05. All investigated variables are presented as mean with 95% confidence intervals (CI) unless otherwise stated. All statistical analyses were performed using Stata (version 14.2; StataCorp, College Station, TX, USA).

In addition, a qualitative analysis of the images was performed for all patients listed in Table 1. The aim of this analysis was to examine pre-to-post changes in the pattern of grouping of specific immune cell types.

## Results

### Adaptive immune response

#### Quantitative assessment

**Fig. 3:**
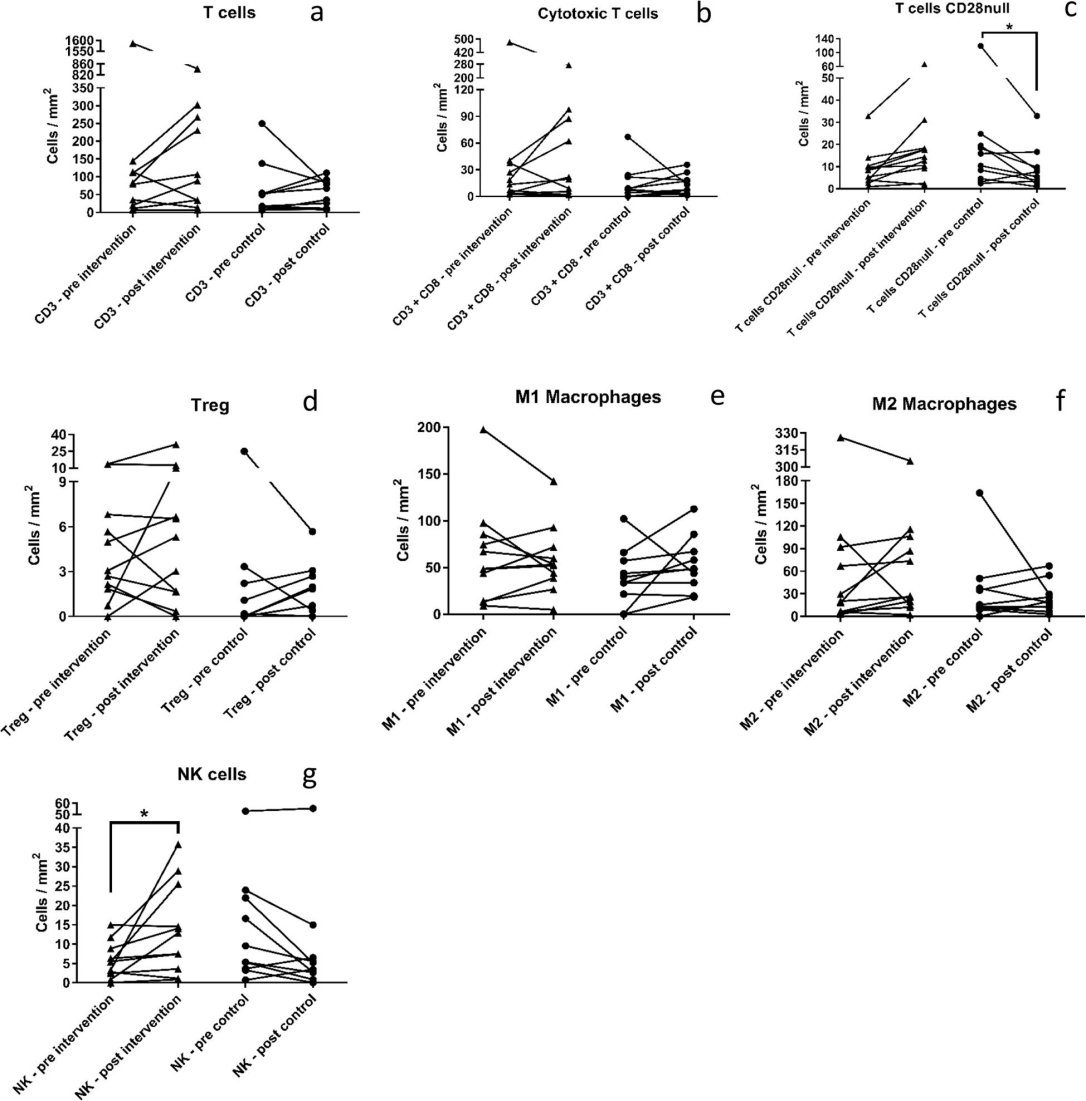
**a** Graph depicting amount of total CD3^+^ T cells. **b** Graph depicting amount of CD3^+^/CD8^+^ cytotoxic T cells. **c** Graph depecting amount of CD28^null^ T-cells. **d** Graph depicting amount of FOXP3^+^ T regulatory. **e** Graph depicting amount of CD68^+^/CD206^−^ M1 macrophages. **f** Graph depicting amount of CD68^+^/CD206^+^ M2 macrophages. **g** Graph depicting amount of CD3^−^/CD8^+^ natural killer cells. Triangles show the pre and post for each patient in the intervention group (*n* = 11). Circles show the pre and post for each patient in the control group (*n* = 10). Bars show group mean. Asterisk signifies a significant change from pre to post (*p* ≤ 0.05). All graphs are shown in cells/mm^2^

The overall number of CD3^+^ T cells, CD3^+^/CD8^+^ cytotoxic T cells and CD8^+^/CD28^null^ cytotoxic subpopulation did not change following the 12-week intervention period in patients allocated to BFRE training or in CON (Fig. 3a, b). In contrast, CD28^null^ expressing T cells (CD3^+^/CD244^+^) decreased from 24.2 (12.4, 36.0) cells/mm^2^ to 10.6 (− 1.1, 22.4) cells/mm^2^ (*p* = 0.032) in CON, while remaining unchanged in BFRE (Fig. 3c), resulting in a between-group difference (*p* = 0.009).

No changes in T_reg_ (FOXP3^+^) expression were noted with BFRE training or in CON (Fig. 3d). T_reg_ cells expressing CD28^null^ averaged 62.6% and 77.5% of the total T_reg_ cell population in BFRE and CON, respectively. No changes were observed following the period of the intervention.

#### Qualitative assessments

**Fig. 4:**
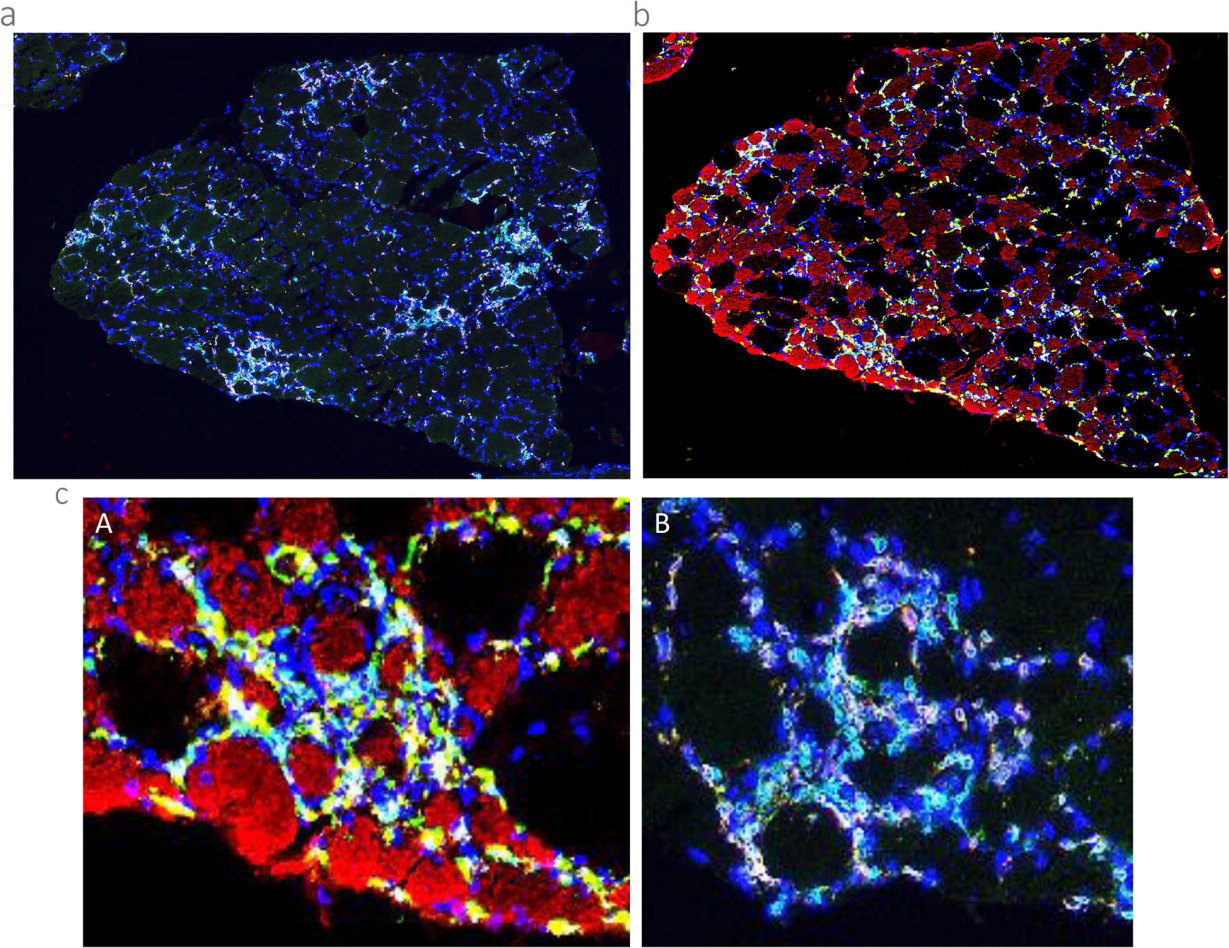
**a** Overview of a T cell staining CD-3 (green), CD-8 (red) and nuclei (Blue). See **b** for a macrophage staining of the same area. **b** Overview of a macrophage staining CD-68 (green), CD-206 (yellow), MHC Fast (red) and Nuclei (blue). See **a** for a T cell staining of the same area. **c** Close up comparison of the same area showing overlapping macrophage and T cell staining around small-sized type 2 fibres. (A) Macrophage staining, with CD-68 (green), CD-206 (yellow), MHC Fast (red) and Nuclei (blue). (B) T cell staining, with CD-3 (green), CD-8 (red) and Nuclei (blue)

No post-intervention changes in the pattern of spatial localisation of CD3^+^ T cells or CD3^+^/CD8^+^ cytotoxic T cells were noted in sIBM patients demonstrating low baseline levels of inflammation. In contrast, patients with high baseline levels of inflammation typically demonstrated an increased frequency of T cell clusters following BFRE training (Fig. 4a). No changes were observed for T_reg_ cells or CD3^+^/CD28^null^ T cells in BFRE except in patients with marked groupings of CD3^+^ T cell infiltration at baseline, in which case clusters of CD3^+^ T cell infiltration tended to also express CD28^null^ (CD244^+^) following BFRE training. No qualitative changes were observed for any T cell-related immune markers in CON from pre to post.

### Innate immune response

#### Quantitative assessment

Pro-inflammatory M1 macrophages (CD68^+^/CD206^−^) and anti-inflammatory M2 macrophages (CD68^+^/CD206^+^) remained unchanged following BFRE intervention as well as in CON (Fig. 3e, f).

NK cell subpopulation (CD3^−^/CD8^+^) density increased following BFRE training from 5.7 (− 2.5, 14.0) to 13.9 (5.6, 22.1) cells/mm^2^ (*p* = 0.003) while no changes were observed in CON (*p* = 0.12) (Fig. 3g), resulting in a between-group difference (*p* = 0.002).

#### Qualitative assessment

M2 macrophages (CD68^+^/CD206^+^) appeared to co-localise more closely to type 2 fibres following the period of BFRE training. Further, several post-intervention biopsies demonstrated signs of clustered macrophage inflammation in the subcellular interstitial space, which was also observed to co-localise more strongly with small-sized type 2 fibres compared to type 1 fibres (Fig. 4b, c).

No change in the localisation pattern of NK cells was observed following BFRE training in sIBM patients with low levels of inflammation and/or few clusters of NK cells at baseline. However, patients with high baseline levels of inflammation and/or high numbers of NK cell clusters typically demonstrated a further increase in NK cell grouping following the period of BFRE training. No qualitative changes were observed in CON from pre to post.

### BFRE intervention—responders vs non-responders

The present data revealed a large within-group variability in T cells, macrophages and in the subpopulation of NK cells, which demonstrating at the histopathological level, myositis patients comprise a highly heterogeneous group. A within-group comparison of patients exposed to BFRE training was performed between patients who showed improvements in all functional test parameters (Timed Up and Go, 30-s Chair Rise, 2-min Walking Test—data previously reported [38]) versus patients who demonstrated a decline in performance in at least two out of three functional test parameters (Table 3).

**Table 3:** Responders vs. non-responders to BFRE training. Responders, patients showing progress in all three tests of functional capacity (FP22, FP15); non-responders, patients with a decline in at least two of the tests of functional capacity (FP3, FP14, FP20). An upregulation (positive response) is defined as a 10% increase or more. A decline (negative response) is defined as 10% decrease or more. No change is defined as a change of less than 10%

Patient	T cells (CD3^+^)			Cytotoxic T cells (CD3^+^ CD8^+^)			CD28^null^ T cells (CD3^+^ CD244^+^)			Cytotoxic T cell subset (CD8^+^ CD244^+^)			M1 macrophages (CD68^+^ CD206^−^)			M2 macrophages (CD68^+^ CD206^+^)		
	Pre	Post	Change	Pre	Post	Change	Pre	Post	Change	Pre	Post	Change	Pre	Post	Change	Pre	Post	Change
FP 22	1689	841	↓	452	24	↓	10	18	↑	74	67	↔	198	142	↓	326	305	↔
FP 15	115	29	↓	31	4	↓	10	10	↔	15	6	↓	98	44	↓	106	15	↓
FP 3	103	243	↑	22	60	↑	9	18	↑	14	51	↑	67	60	↓	67	73	↔
FP 14	3	4	↑	0.4	0.0	↓	3	13	↑	6	9	↑	13	39	↑	3	20	↑
FP 20	10	29	↑	3	6	↑	16	32	↑	10	11	↔	49	53	↔	6	27	↑

As illustrated in Table 3, patients (FP 22/15) with uniform improvements in functional capacity demonstrated uniform changes in the composition of their inflammatory profile. The only noticeable exception was for M2 macrophage’s response, where FP22 showed a minor decrease (< 10%) following BFRE training. In contrast, patients demonstrating a general decline in functional capacity in response to BFRE intervention (FP 3/14/20) showed a more variable and less uniform pattern of change in inflammatory profile. Specifically, T cells increased more than 10% for these patients (FP 3/14/20) while cytotoxic T cells increased in two out of three patients. Changes in M2 macrophage content exceeded 10% in two out of the three patients with concurrent decline in functional capacity (FP 3/14/20) (Table 3).

Comparing BFRE-trained responders to non-responders, a number of notable trends were observed. As an overall pattern, T cells, cytotoxic T cells, the cytotoxic subpopulation and M2 macrophages seemed to change reciprocally with changes in functional status, demonstrating decreasing values in those patients who improved their functional capacity, while increasing in patients with a decline in performance. Notably, a majority (4/5) of BFRE trained patients showed a ≥ 10% increase in CD28^null^ T cells following the period of BFRE training.

## Discussion

The main finding of the present study was that 12 weeks of BFRE training led to an increased number of CD3^−^/CD8^+^ NK cells in sIBM patients, resulting in a between-group difference when compared to untrained sIBM controls. In contrast, CON patients (no training) showed a decrease in the number of infiltrating CD28^null^ T cells (CD3^+^/CD244^+^), resulting in a between-group difference. No longitudinal changes in cytotoxic T cells (CD3^+^/CD8^+^), regulatory (FOXP3^+^) T cells and M1 and M2 macrophage density were observed following BFRE as well as in CON.

Notably however, qualitative analysis indicated that the spatial positioning of M2 macrophages was altered following BFRE training, as manifested by a preferential accumulation of M2 macrophages around small-sized (possibly atrophied) type 2 fibres.

Macrophage and T cell densities are known to be high in sIBM patients [1, 22], which was supported by the present observations. The elevated levels of macrophages and T cells at baseline may have affected the present BFRE training response, while also contributing to explain the general lack of improvements in physical function in the present group of BFRE-trained sIBM patients [21]. Something which has otherwise been observed to improve following similar type of training in PM and DM patients [20], who demonstrated low levels of macrophages and T cells, both at baseline and following training intervention [40, 41].

### Adaptive immune response

Previous research on immune system function in IIM patients has mainly been focused on T cells. In the recent years, CD28^Null^ T cells have become an increasingly important marker for cytotoxicity and indicator of reduced immunological plasticity in IIM patients, as well as being a highly discussed topic in clinical immunology [22–24, 42–44].

High levels of CD28^Null^ T cells have been associated with elevated cytotoxicity, impaired immune system plasticity and reduced capacity for T cell proliferation [25, 26, 45]. Interestingly, the present study demonstrated a longitudinal loss of CD28^Null^ T cells in the non-trained CON group. At first hand, these data would seem to suggest that refraining from exercise-based physical activity might have a positive effect on immune system function in sIBM patients. In disfavour of this notion, however, BFRE training did not lead to any increase (nor decrease) in CD28^Null^ T cell content in the present group of sIBM patients. While increasing abundance of CD28^Null^ T cells have been associated with progressive ageing [46–48] and inflammatory myopathic disease progression [42], no longitudinal changes in cytotoxic T cell markers (CD3^+^/CD8^+^, CD8^+^/CD28^Null^) were observed in the present study. Collectively, these observations suggest BFRE training to not have any major detrimental effect on the immunological muscle milieu in sIBM patients.

Previous studies in healthy adults have reported that physical exercise upregulates T_reg_ cells in peripheral blood, acutely following exercise [49, 50]. Consequently, we initially expected that low-load BFRE training would lead to an upregulated number of T_reg_ cells in our group of sIBM patients. However, no change in T_reg_ cell content was observed post intervention. This lack of expected change may have been due to the fact that the present sIBM patients demonstrated a high abundance of CD28^null^ T cells already prior to training, including high levels of FOXP3^+^/CD244^+^ (T_reg_/CD28^null^) cells that are known to be elevated with increasing age and prolonged disease duration in sIBM patients [25, 26, 45]. The population of CD28^null^ T cells have been shown to be functionally active, long-lived, oligoclonal lymphocytes that lack or have limited proliferative capacity regardless of subtype allocation [25, 26, 45].

sIBM patients have lower amounts of activated peripheral T_reg_ cells compared to age-matched controls [33]. Animal (mice) experiments have demonstrated that a T_reg_-deficient muscle milieu (reduced amounts of T_reg_) combined with aberrant muscle antigen exposure allows spontaneous myositis to occur [51]. Further, it has been reported that the functional suppressive effect of T_reg_ cells on the proliferation of autologous T cells is not impaired in sIBM patients compared to healthy age-matched controls [33]. Thus, it may be speculated that the global (muscle and circulating) decline in T_reg_ cell abundance at least in part accounts for the impaired muscle regeneration/re-building capacity uniformly observed in sIBM patients. Thus, it becomes of interest to investigate if BFRE training intervention in younger and/or more newly diagnosed sIBM patients will lead to an upregulation in activated and/or non-activated T_reg_ cell content. If documented in futures studies, this could indicate an early-phase responsiveness to anabolic exercise that would emphasise the importance of early diagnosis in sIBM patients, which would allow early exercise-based rehabilitation to be initiated.

### Innate immune response

High-frequent (twice daily) BFRE training [16] as well as exercise in general [52] appear to promote an anti-inflammatory macrophage response in healthy young adults. In contrast, no quantitative changes in macrophage content were detected following BFRE training in the present group of sIBM patients.

In skeletal muscle tissue, the transition from a M1- to a M2-dominated milieu is considered supportive for muscle regeneration and has been functionally coupled with myogenesis [32]. However, given that our sIBM patients demonstrated an abundance of both M1 and M2 macrophages prior to training, this might have attenuated or blunted the expected training-induced transition from the M1 towards M2 macrophage state. Thus, potentially resulting in an impaired muscle regeneration response when training. Further, the low and invariant numbers of T_reg_ cells presently observed could also account for some of the attenuation of the transition of M1 towards M2, as T_reg_ cells are important for this transition to occur [32, 53].

When comparing the spatial co-grouping of small-sized possibly atrophied type-2 fibres with extensive macrophage infiltration to the corresponding myocellular regions stained for T cells and NK cells, a high degree of overlap was noted. Specifically, the grouping of small-sized type 2 myofibres systematically coincided with high levels of macrophage infiltration (both M1 and M2), accompanied by high levels of T cells (CD3^+^), cytotoxic T cells (CD3^+^/CD8^+^) and the specific NK cell subpopulation (CD3^−^/CD8^+^) (Fig. 4b, c).

These observations indicate a concerted immune response mainly targeting small-sized type 2 (atrophied) fibres. Macrophages have recently been reported as being able to obtain immunological memory that affects their responsiveness to subsequent acute tissue injury [54], which may resemble the ongoing conditions in sIBM patients due to their state of chronic inflammation.

The present study revealed a substantial upregulation in the expression of the investigated NK cell subpopulation following the BFRE intervention (Fig. 3c). At first glance, this observation might be interpreted as a contraindication for the use of BFRE in sIBM patients, as NK cells are known to be of cytotoxic nature [55]. However, it remains unclear of which magnitude NK cell upregulation is required for it to become detrimental to sIBM patients. For example, acute exercise in healthy adults leads to acutely upregulated NK cell number and activity, respectively [36, 56]. Further, the level of NK cells appears to be higher in elderly competitive endurance athletes compared to sedentary age-matched controls [57].

sIBM patients are characterised by high levels of inflamed muscle tissue, and therefore, it could be argued that an elevated NK response would be interpreted as a sign of worsening of their disease state. However, all responding and non-responding sIBM patients demonstrated an upregulation in NK cells irrespectively of their performance in the physical function tests (Table 3), indicating that elevated levels of NK cells are likely not detrimental for functional capacity. Therefore, further investigation of NK cell content and their role in muscle homeostasis should be conducted.

### Methodological considerations

In the present study, paired (pre/post intervention) biopsies from 16 sIBM patients were analysed. To our best knowledge, this is the first report on histological changes in the skeletal muscles of sIBM patients undergoing BFRE training.

The present within-subject design (pre vs. post training biopsy comparisons for each individual study participant) reduced the potential influence of large inter-individual variations on the statistical analysis outcome. In support of this approach, a large magnitude of inter-individual heterogeneity was observed in the present histological data, although the corresponding clinical and functional data showed a more homogenous picture of this patient group [38]. This questions (I) whether sIBM patients can be considered a homogeneous subject group and (II) if the temporal development of the disease alters patients’ responsiveness to specific types of interventions, such as low-load BFRE training.

Reproducibility is an issue in histopathology in general and has also been dealt with in the case of inflammatory myopathies (mainly IIM) [58], where a broad range of sensitive cellular and histological markers have been suggested by various expert panels. Disagreement on both diagnostic criteria and severity of histological changes seem to exist in the literature, yet reproducible data on severity is important, particularly when it comes to studies on effects of intervention. To overcome this problem the present study employed automatized counting of inflammatory cells, to eliminate inter-observer variance. Our setup allowed detection of co-localisation for cell subclassification, but it would be of interest in future analysis to also include data on topographical distribution parameters, muscle damage, regeneration and vascular properties.

A number of potential limitations may be listed for the present study. Due to the rarity of the disease, a relatively low number of sIBM patients were recruited for the study. Further, a substantial amount of heterogeneity was observed in terms of age, disease duration and medical treatment history [38], which likely explain the corresponding heterogeneity in histopathology among the present group of sIBM patients. Further, no healthy age-matched individuals were included, since the overall objective was to investigate the effect of BFRE training on immune system function in sIBM patients. Consequently, it was not possible to adjust the present data for the natural time course of age-related changes. Finally, one patient dropped out of the study due to severe fatigue.

## Conclusion

Blood-flow restricted muscle exercise (BFRE) represents a novel approach in the treatment and/or rehabilitation of sIBM patients. The findings from this study suggest that BFRE training is a feasible and tolerable training regime for sIBM patients, since no detrimental or adverse effects on immune system function could be noted following a 12-week training intervention. Further research into the design of specific BFRE training protocols for sIBM patients is needed. In addition, research efforts into the effect of BFRE training on regenerative myocellular markers including stem cell (satellite cell) proliferation and capillary neoformation also seem warranted to gain a deeper understanding of the potential benefits of BFRE intervention in this frail patient population.

## Data Availability

Data is not available due to current GDPR regulations.

## Abbreviations

BFRE: Blood flow restriction exercise
CON: Control group/non-exercising control
DM: Dermatomyositis
IIM: Idiopathic inflammatory myopathy
M1: Pro-inflammatory macrophage
M2: Anti-inflammatory macrophage
NK: Natural killer
PBS: Phosphate-buffered saline
PM: Polymyositis
sIBM: Sporadic inclusion body myositis
